# A qualitative synthesis of diabetes self-management strategies for long term medical outcomes and quality of life in the UK

**DOI:** 10.1186/1472-6963-14-348

**Published:** 2014-08-16

**Authors:** Julia Frost, Ruth Garside, Chris Cooper, Nicky Britten

**Affiliations:** Institute of Health Research, University of Exeter Medical School, Veysey Building, Salmon Pool Lane, Exeter, EX2 4SG UK; European Centre for Environment and Human Health, University of Exeter Medical School, Knowledge Spa, Royal Cornwall Hospital, Truro, TR1 3HD UK; Peninsula Technology Assessment Group (PenTAG), University of Exeter Medical School, Veysey Building, Salmon Pool Lane, Exeter, EX2 4SG UK

**Keywords:** Diabetes, Biomarkers, Synthesis, Meta-ethnography, Self-management, Longitudinal, Qualitative, Quality of life

## Abstract

**Background:**

Qualitative research on self-management for people with Type 2 Diabetes Mellitus (T2DM) has typically reported one-off retrospective accounts of individuals’ strategies. The aim of this research was to identify the ways in which self-management strategies are perceived by people with T2DM as being either supportive or unsupportive over time, by using qualitative findings from both longitudinal intervention studies and usual care.

**Methods:**

A systematic review of qualitative literature, published between 2000 and 2013, was conducted using a range of searching techniques. 1374 prospective qualitative papers describing patients’ experiences of self-management strategies for T2DM were identified and screened. Of the 98 papers describing qualitative research conducted in the UK, we identified 4 longitudinal studies (3 intervention studies, 1 study of usual care). Key concepts and themes were extracted, reviewed and synthesised using meta-ethnography techniques.

**Results:**

Aspects of self-management strategies in clinical trials (e.g. supported exercise regimens) can be perceived as enabling the control of biomarkers and facilitative of quality of life. In contrast, aspects of self-management strategies outwith trial conditions (e.g. self-monitoring) can be perceived of as negative influences on quality of life. For self-management strategies to be sustainable in the long term, patients require a sense of having a stake in their management that is appropriate for their beliefs and perceptions, timely information and support, and an overall sense of empowerment in managing their diabetes in relation to other aspects of their life. This enables participants to develop flexible diabetes management strategies that facilitate quality of life and long term medical outcomes.

**Conclusions:**

This synthesis has explored how patients give meaning to the experiences of interventions for T2DM and subsequent attempts to balance biomarkers with quality of life in the long term. People with T2DM both construct and draw upon causal accounts as a resource, and a means to counter their inability to balance medical outcomes and quality of life. These accounts can be mediated by the provision of timely and tailored information and support over time, which can allow people to develop a flexible regimen that can facilitate both quality of life and medical outcomes.

## Background

By 2030, 500 million adults worldwide will have diabetes, with 2.5 million predicted in the UK [1]. UK policymakers have described the burden associated with the progressive nature of diabetes in terms of direct costs to the NHS and associated healthcare support services; indirect costs to the economy due to loss of productivity; and the personal impact of diabetes, and complications for patients and their families [2]. The National Service Framework for Diabetes called for a ‘skills-based approach [to] support self-care by improving knowledge, blood glucose control, weight and dietary management, physical activity and wellbeing’ ([3]:16); and the associated NICE guideline for Type 2 Diabetes Mellitus (T2DM) recommends that people with diabetes should be offered structured education [4]. However, a review of service organisation and delivery in the UK identified a lack of health services research in diabetes; variability in the quality and range of support that is provided to people with diabetes; and a recognition that supporting self-care behaviours is challenging for many groups of patients with diabetes [5].

Randomised controlled trials of educational interventions, specifically for T2DM in the UK, have proved inconclusive. A trial of an expert patient education intervention found that it improved glycaemic control, reduced total cholesterol level, body weight, BMI and waist circumference, reduced requirement for diabetes medication, increased consumption of fruit and vegetables, enjoyment of food, knowledge of diabetes, self-empowerment, self-management skills and treatment satisfaction; but there was no overall improvement in quality of life at fourteen months [6]. A trial of diabetes education and self-management for ongoing and newly diagnosed (DESMOND) programme found that while those in the intervention group reported positive improvements in beliefs about their illness, there was no difference in HbA1c or dimensions of quality of life at twelve months [7]. Furthermore, a recent systematic review established that tailored interventions for T2DM, hypertension and heart disease had no impact on medication adherence, self-monitoring, exercise, smoking or diet control, while having a modest impact on screening, dietary fat intake and levels of physical exercise [8].

An observational study found that people with T2DM rationalise their understanding and response to diabetes by externalising control of their condition to health care professionals who are responsible for their care [9]; while a qualitative study with participants from the DESMOND trial identified that individual orientations (degrees of acceptance or resistance to either their new ‘diabetic’ identity or any perceived consequences of that identity, and perceptions of the required degree of personal responsibility) may mediate both education preference (e.g. group education that is peer or professional led) and ultimately self-management [10]. While both provide useful insights, they only collected retrospective accounts via one-off interviews [11]. Given the long term, chronic nature of T2DM this may not capture aspects of change or development in attitudes and behaviours over time.

Existing syntheses of qualitative research (including only non-intervention studies) indicate that patients with T2DM often prioritise the maintenance of their current quality of life over future improvements to their biomarkers. Paterson et al. demonstrated that people learn to balance their diabetes by combining experience with experimenting with strategies for managing their illness [12]; while Campbell et al. identified that balance required a complex process of understanding and ‘an ability to manipulate dietary and medication regimens in order to live life as fully as possible, rather than limiting social and work activities in order to adhere rigidly to medical advice’ ([13]:681). More recently, Gommersall et al. have established the salience of culture and gender roles, as well as perceptions of threats to selfhood [14]. However, we are not aware of any syntheses that have explicitly compared observational and intervention-linked longitudinal qualitative studies of diabetes self-management. Such a synthesis could illuminate the long term sequelae of diabetes self-management strategies, and prove crucial for understanding the impact of the progressive and degenerative nature of diabetes [15, 16].

The aim of this qualitative synthesis was to identify the ways in which self-management strategies are perceived by people with T2DM as being either supportive or unsupportive, from prospective qualitative research using data collected on two or more occasions over a twelve month period, and to compare experiences of those taking part in intervention studies and those receiving usual care.

## Methods

Systematic reviews use a structured approach to identify, appraise and synthesise research [17]. Here the meta-ethnographic method described by Noblit and Hare was used to conduct this qualitative synthesis [18]. Meta-ethnography is an interpretive rather than an aggregative approach, which involves the translation of individual qualitative studies into each other, through the re-interpretation and transformation of their theoretical and substantive concepts [13, 16, 19]. We adhered to the enhancing transparency in reporting the synthesis of qualitative research (ENTREQ) statement [20].

A database search (MEDLINE, EMBASE, Social Policy and Practice, HMIC and PsycINFO, all via OVID) was conducted in February 2013 using terms for diabetes/diabetics (diabet*) combined with a qualitative research methods literature search filter written by the Health Information Research Unit, McMaster University strategy [21], In addition:

- Meta-syntheses of diabetes qualitative research were identified and forwards and backwards citations found;
- Key authors were identified and their papers were located and screened;
- Key trials were citation chased and searches were made using the trial acronyms as search terms;
- Experts in the field were contacted for published and unpublished papers.

References, including titles and abstracts, were then loaded into Endnote X5 [22], and the search was updated in May 2013.

Papers were included at the title and abstract screening stage [by JF] if they:

- Were among adults with T2DM,
- Conducted in the UK (reflecting current practice in the National Health Service),
- Published since 2000.

Full text papers were obtained and were further screened [by RG, NB] to establish if they:

- Used recognised qualitative methods of data collection and analyses,
- Used longitudinal qualitative data collection – with two or more periods of data collection
- Used follow–up qualitative data collection of at least 12 months.

We appraised the quality of the included papers using a validated appraisal tool [23] as a means to assess the rigour and validity of the data collection and analysis techniques employed by the research authors (Table 1), which informed our analysis of the data [24].

**Table 1:** **Quality appraisal of included qualitative papers and associated quantitative papers**

	DALY [25–27]	EarlyACTID [28–30]	PACCTS study [31–37]	Usual care study [11, 38–45]
**Methods**	Convenience sample: All patients with T2DM participating in an RCT (n = 89; 53 in the intervention arm, 36 in the control group)	Purposive sample: 30 patients with newly diagnosed T2DM participating in an RCT. Sampled to represent trial arm, recruitment site, and gender.	Purposive sample: 25 participants representing 4 groups depending on HbA1c control: ‘good’ (<7) or ‘poor’ (>9) (n = 13); or ‘improving’ or ‘deteriorating’ (n = 12)	Convenience sample: 40 patients with newly diagnosed T2DM.
Sample				
Diabetes duration at baseline (mean)	6 years	6 months	6 years	6 months
Data collection	Cycles of semi-structured focus groups with all trial participants, pre- (n = 5) and post-intervention (n = 5). Group A at 6 and 12 months; Group B at 6 and 18 months; Group C at 12 months	Face to face interviews at 6 months (n = 30), and follow -up telephone interviews at 9 months (n = 29).( Trial paper [29] has 12mth data)	Semi structured face-to-face interviews at12 months (n = 25) ,and 24 months (n = 11).9 matched consultation sessions and telephone interviews at 36 months.	Semi structured face-to face interviews and fieldnotes at 0, 6, and 12 months (n = 40) and 48 months (n = 20).
Analysis	Constant comparison. Source, method and theoretical triangulation.	Constant comparison. Thematic analysis.	Constant comparison. Thematic analysis. Construction of extended case reports over time.	Grounded theory [38, 39]; Thematic analysis [40, 45]; Longitudinal [11];
Setting	Primary and secondary care, England	Primary care, South West England	Primary care, deprived area in North West England.	Primary and secondary care, Scotland
Trial design	89 patients, randomised control wait list design Group A were randomly allocated to the treatment initially (n = 30), whilst the Group B acted as the short-term control group (n = 23) These two groups were then combined to form the short term trial group.	593 patients randomly assigned in a 2:5:5 ratio. Control n = 99, Intensive Diet n = 249, Diet plus Activity n = 246	591 patients randomly allocated in a1:2 ratio. Control n = 197, Call-centre treatment support n = 394.	N/A. 40 patients with T2DM. Explorations of variance, location of care (12.5% primary care, 87.5% secondary care), diet, medication, class and gender.
	Group C received the intervention at the end of the trial period (n = 36).	Patients randomized to ‘usual care’ received standard advice about diet from trial dieticians at their baseline visit, and were seen by a doctor blinded to treatment at baseline, six and twelve months [28].	Patients randomized to the ‘usual care’ group continued with conventional treatment based on local guidelines, which had been in place for over ten years, supported by a continuing education program among all primary care practices [34].	
Intervention	8 week educational programme including: physical activity, exercise, relaxation and health topics.	Intensive diet (ID) or intensive diet plus activity (IDPA)	Tele-care phone support, titrated to HbA1c, to improve blood glucose control	Not applicable
Trial results	At 6 months, intervention associated with benefits in HbA1c levels (−0.1%), illness attitudes, and perceived treatment effectiveness, compared to controls. At 12 months, only illness attitudes and self-monitoring showed benefit [27].	At 6 and 12 months, glycaemic control had improved in the diet (−0.28%) and diet/activity groups (−0.33%), but worsened in the control group [29]	At 12 months, compared with the control group, HbA1c improved by 0.31% in the intervention group, and the improvement was significantly greater for those with a baseline HbA1c > 7% [30].	Not applicable
		At 12 months, the control group saw an improvement in their understanding, expectation of disease continuation, and concern of their illness; while the intervention groups increased their understanding, became less concerned, felt more in control of their illness, were more satisfied with their diabetes treatment, and had higher self-reported health scores [30].	At 12 months, the intervention group continued to report high levels of satisfaction with their treatment [34]	
			At 36 months, there was a statistically significant reduction of HbA1c by 0.24% attributable to the intervention [36].	

The research team repeatedly read the studies to identify key themes and concepts, and to identify areas of consonance and dissonance between the included studies. JF produced a structured summary for each paper, and with input from RG and NB, tabulated the key results, concepts and themes for each study, so that they could be compared and contrasted. Quotations from patients and carers (first order data), as well as author interpretations of the data (second order interpretations) were interpreted and integrated by the research team (into third order concepts) to produce a ‘line of argument’ [13, 18, 19].

The purpose of meta-ethnography is to identify where similar concepts and themes from different studies or papers refer to the same entity (congruent synthesis) or to opposing findings (refutational synthesis); this is referred to as the papers’ findings being ‘translated’ into each other [18, 46, 47]. Thus, as interpretations emerged, they were subject to systematic testing within and between the studies, and in consultation with a wider stakeholder group. This group included People with Type 1 or Type 2 Diabetes, members of the South West Peninsula Diabetes Research Network, Diabetologists, General Practitioners (with and without a special interest in diabetes), the Director of Public Health, and community or hospital based Diabetes Specialist Nurses.

## Results

**Figure 1:**
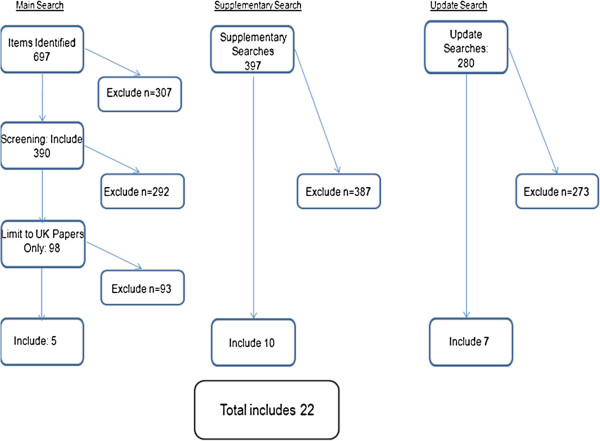
**Search flowchart.** A database search was conducted using terms for diabetes and qualitative research methods, Additional methods included citation chasing and key author/paper identification. Papers were included at the screening stage if they were among adults with T2DM; conducted in the UK; and published since 2000. Full text papers were obtained and screened to establish if recognised longitudinal qualitative methods of data collection and analyses were used.

The searches resulted in 1374 abstracts (Figure 1). Preliminary screening identified that 98 of these papers described qualitative research conducted in the UK. A full text screen identified only four studies (reported in 15 papers) that used longitudinal data methods over 12 months or more. An updated search in May 2013 identified 280 further abstracts of which seven conference abstracts about the four studies were identified. Similar to a meta-ethnography of patients’ experience of managing anti-depressants we identified two groups of papers early in the synthesis process and grouped them along a timeline [48]. Reflecting the growing use of longitudinal qualitative methods in medical research more generally, the first group concerned serial qualitative studies that were nested within randomized controlled trials of interventions in order to elucidate causal pathways, while the second were stand-alone studies exploring the influence of health services on the conceptualization of illness over time [49].

Six papers concerned three studies where qualitative methods were nested in trials of self-management interventions. The DALY study employed focus groups to explore the impact of an eight week educational programme administered to all trial participants (n = 89) via five trial courses running over a period of one year [25, 26]. The Early ACTID study purposively sampled participants from both trial arms and various recruitment sites to elicit patient perspectives of a trial of diet versus diet and exercise versus usual care via face to face and telephone interviews [28]. The PACCTS study purposively sampled participants from a deprived urban area, and allocated them to four study groups (where HbA1c control was deemed to be ‘good’, ‘bad’, ‘improving’ or ‘deteriorating’), in order to determine the utility of a tele-care support intervention that was titrated to HbA1c results [31–33]. Although the qualitative data collection in the paper by Malpass et al. was only over a period of nine months [28], its findings were substantiated by data in an associated quantitative paper that extended over twelve months [29].

Nine papers were about a single sample of patients, recruited from both primary and secondary care within six months of diagnosis with Type 2 Diabetes, and concerning their perceptions of self-management in the context of changes to diabetes service delivery in Scotland. Four papers utilised interviews at baseline six months and 12 months [38–41]; and five papers providing additional data collected at 48 months [11, 42–45] (Table 2). This set of papers provided a rich data set, which variously employed grounded theory, thematic analysis and longitudinal data analysis techniques to interrogate the sixty interviews, in terms of the patients’ perspectives of service delivery [39] as well as discrete concepts such as control and causality [42, 43].

**Table 2:** **Profile of studies**

	Is the research question clear?	Perspective of author clear?	Perspective influenced the study design?	Is the study design appropriate?	Is the context adequately described?	Sample adequate to explore range of subjects/ settings?	Sample drawn from appropriate population?	Data collection adequately described	Data collection rigorously conducted?	Data analysis rigorously conducted?	Findings substantiated/limitations considered?	Claims to generalisability follow from data?	Ethical issues addressed?
Cooper 2003 [25]	Y	Y	Y	Y	N	Y	Y	Y	Y	Y	Y	Y	Y
Cooper 2003 [26]	Y	Y	Y	Y	N	Y	Y	Y	Y	Y	CNT	Y	Y
Malpass 2009 [28]	Y	Y	Y	Y	Y	Y	Y	Y	Y	Y	Y	Y	Y
Gambling 2010 [31]	Y	Y	Y	Y	N	CNT	Y	Y	Y	Y	Y	Y	Y
Gambling 2010 [32]	Y	Y	Y	Y	Y	Y	Y	Y	CNT	CNT	CNT	Y	Y
Long 2011 [33]	Y	Y	Y	Y	C	CNT	Y	Y	Y	CNT	Y	Y	N
Lawton 2004 [38]	CNT	Y	CNT	Y	Y	Y	Y	Y	Y	Y	CNT	CNT	CNT
Lawton 2005 [39]	Y	Y	Y	Y	Y	Y	Y	Y	Y	Y	N	Y	Y
Lawton 2005 [40]	Y	Y	Y	Y	Y	Y	Y	Y	Y	Y	N	Y	Y
Parry 2006 [41]	CNT	Y	Y	Y	Y	Y	Y	Y	Y	Y	CNT	Y	Y
Peel 2007 [42]	Y	CNT	CNT	Y	Y	Y	Y	Y	Y	Y	N	Y	Y
Lawton 2008 [43]	Y	Y	Y	Y	Y	Y	Y	Y	Y	Y	N	Y	Y
Lawton 2008 [44]	Y	Y	Y	Y	Y	Y	Y	Y	Y	Y	CNT	Y	Y
Lawton 2009 [45]	Y	CNT	Y	Y	Y	Y	Y	Y	Y	Y	Y	Y	Y
Peel 2010 [11]	Y	CNT	CNT	Y	Y	Y	Y	Y	Y	CNT	Y	N	Y

Key: Y=Yes, N= No, CNT = Cannot tell.

The inclusion of these papers allowed the reviewers to explore any potential influence of either trial design (e.g. a supportive trial environment may enhance the positive impact of the interventions) or study duration (e.g. ongoing information and support, which are central to the underpinning philosophies of the intervention studies, and foster understanding and confidence, and ultimately an enduring self-efficacy). Combining studies with diversity of purpose provides opportunities for comparisons and potentially more fruitful and meaningful insights, than that which may be obtained by synthesising papers that are more obviously methodologically or substantively ‘similar’ [50].

Using the established synthesis techniques outlined above, the reviewers identified and translated the key concepts in each of the papers (e.g. the individual study authors’ second order interpretations of the ‘raw’ or ‘first order’ patients’ words) into three interlocking third order constructs (Table 3, first column): Patient as stakeholder (a patient actively engaged with their service provision), Timeliness of support (the appropriate provision of provision and support, often repeatedly, tailored to the current needs and preferences of the patient, and their subjective understanding of their condition), and Empowerment (the willingness and ability of people with diabetes to self-manage in order to achieve purposive and meaningful behaviour change strategies); and from these produced a line of argument about the support needs of people with T2DM (Table 3). Subsequent columns in Table 3 summarise the second order concepts, and the narrative ‘translation’ of those concepts, and details of the papers from which they were drawn, which were listed and tabulated so that they could be explored, compared and juxtaposed.

**Figure 2:**
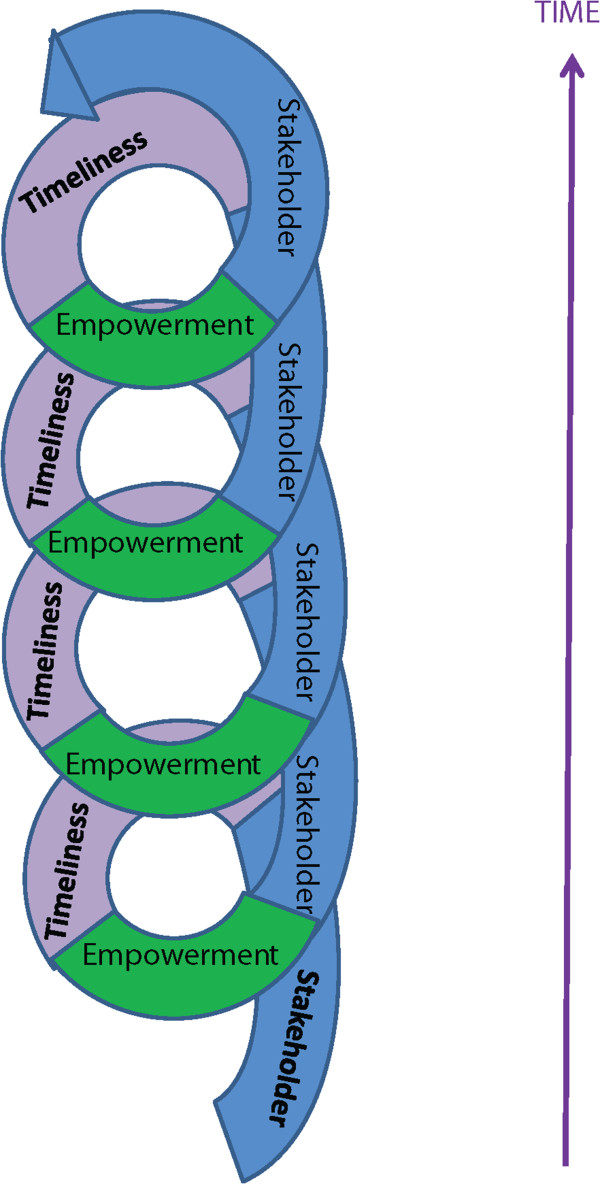
**Three interlocking concepts.** This qualitative synthesis identified three interlocking third order constructs (Patient as stakeholder, Timeliness of support, and Empowerment), and a line of argument that stated that for self-management strategies to be effective, people with diabetes require a sense of ownership of the management of their disease. This can be fostered through the timely provision of information and advice that acknowledges and accounts for their individual circumstances (e.g. disease duration, and prior experience of diabetes management).

We conceive of these three aspects as interlinked and supporting each other, and there is some overlap, but they represent the cornerstones of good support that allow people with T2DM to successfully manage their condition. These aspects can be mutually supporting, leading to a virtuous circle and improved self-management, while their absence may lead to a vicious circle for patients as they become increasingly disempowered, unable to participate in their own self- care and unable to access the support they need to change (Figure 2).

**Table 3:** **Translation of second order constructs**

Third order construct	Second order constructs	Summary definition of the second order construct(s)	Papers that include the second order constructs
Patient as stakeholder	Building a picture	Patients respond better to advice that is tailored to their needs –but staff do not always do this.	[25; 28; 31; 38; 39]
	Personalised advice		
	Appropriateness		
	Meaning/Understanding		
	Sharing and finding common ground	Patients feel ownership when their views and experiences are valued - but staff attitudes can undermine this.	[25; 26; 32; 38; 39]
	Ownership		
	Resource allocation	Patients value sustainable support and information provision - but this is resource intensive	[27; 28; 34; 37; 48; 51]
	Resource use		
Timeliness	Timeliness	Patients benefit from having gaps in their knowledge addressed at their own pace (e.g. if they can ask fundamental questions beyond the initial assessment) – without these opportunities lay interpretations develop.	[25; 31; 35; 42]
	Access		
	Phased approach		
	Contextual knowing		
	Consciousness raising	Patients value having information and support that matches their current perspective (e.g. if/when they are ready to understand their responsibility) – otherwise patients can disengage with service provision and/or self-management.	
	Aligning patients’ needs		
	Responsive advice		
	Implementing a sustainable plan	Patients are motivated to change their behaviour, when practices are perceived as improving their quality of life –but suggestions from staff that are perceived as impairing quality of life can be perceived of as out of touch with reality	[11; 26; 28; 32; 45]
	Self-management behaviours		
Empowerment	Empowerment	With understanding, some patients are able to develop a flexible regimen (e.g. titrate exercise to treats and cheats) – but without ongoing support some do not develop appropriate causal models.	[25; 26; 28; 35; 36; 37; 41; 42; 45]
	Down to me/Up to them		
	Sustainability	Flexible regimens can enhance both control of blood glucose and quality of life – but without tailored/ ongoing education that goes beyond ‘learning by rote’ many patients find this difficult to achieve.	[28; 32; 34; 35; 36; 43; 44; 45]
	Commitment		
	Accounts as resources		
	Forgetfulness

### Patient as stakeholder

By comparing and synthesising the key concepts in the intervention and usual care studies, a third order concept was developed about the notion of patient as a stakeholder: a patient actively engaged with their service provision. Participants in the intervention studies were strategically encouraged to ‘own’ and actively manage their diabetes, while those in receipt of usual care did not have the advantage of benefiting from the intervention, nor of staff with additional expertise. In its absence they provided their own interpretation of both their condition and their role in its management.

Within the trial of an educational support intervention (PACCTS) self-management was fostered by tele-carers tailoring information to individuals’ needs [28]. An example is provided of a woman who had had a stroke, where information was provided to her husband to optimise ‘portion control’ [Participant quote, [31]: 183] at mealtimes, as exercise was not a viable weight-reduction strategy. This advice was subsequently reinforced by the tele-carer acknowledging that weight loss is difficult (using ‘empathy’) and encouragement to the couple to maintain their efforts (‘positive reinforcement’) [Author quotes, [31]: 183].

Participants receiving an eight week diabetes educational intervention (DALY) similarly valued receiving information that was tailored to their circumstances, in contrast to their previous experience of care:

One participant described the course as an ‘eye-opener’, whilst another said, ‘I’ve learnt more in the first hour here than I’ve learnt in nearly 5 years’. The course provided participants with the details of managing their disease within the context of their everyday lives, with frequent references to learning about ‘individual’ and ‘small things’ [Author quotes, [25]: 199]

Participants suggested that having nurse tutors who showed ‘integrity, respect and compassion toward them, as well as demonstrating their nursing expertise in diabetes’ fostered a ‘process of moral interaction’ [Author quotes, [25]: 200]. This facilitated patient participation in both their education, and development of the skills for effective self-management, while nurturing reciprocal health care relationships which are fundamental to ‘quality of life for people with chronic illness’ [Author quote, [25]: 200].

Respect and reciprocity were also viewed as fundamental precursors to enabling patients to be honest about their ability and desire to self-manage, deemed essential by the PACCTS study authors, for planning sustainable behaviour change beyond the duration of a trial. The tele-carers in the PACCTS study acknowledged that if patients were too regimented in their routines there was the potential to ‘fall off the bandwagon’ [Tele-carer quote, [32]: 223]; while the participants’ narratives demonstrated how some felt able to try out new management strategies and modify them to fit into their daily routines, and report this to a non-judgmental professional:

‘Other things we discussed were regular food intake. I felt that she really wanted me to have my breakfast at 8, lunch at 12, tea at 4. But I explained that I can not do that. Even when you are getting older, you still have your own way of doing things. We had a discussion about the gap. I would have high readings at lunchtime and she wanted to know the cause. It was probably because I was trying to comply with her regulations but I was not getting out of bed until late, finishing my breakfast 9:30 or 9:45 and then having dinner at 1. So, we decided not to do it like that.’ [Participant quote, [32]: 224]

In contrast, reflections on ‘usual care’ from participants in the health education study (DALY) reported that health professionals did not always embrace participants’ self-perceived needs, and talked about the difficulties they had in getting professionals to respond in what they considered to be an appropriate fashion (e.g. when patients transferred from oral therapies to insulin). The study authors conclude that ‘the lack of an appropriate response by health professionals highlighted the interaction of factors that can affect the clinical outcomes associated with educational trials’ [Author quote, [26]:49]

However, while facilitating patient participation in the management of their diabetes, these interventions and trial environments are resource intensive. All of the participants in the EarlyACTID trial had a consultation with a doctor at baseline, six and twelve months, while those in the intervention arms also received:

‘fifteen nurse or dietician visits of twenty minutes each over a twelve month period …seeing the same nurse or dietician throughout the trial.’ [Author quote, [28]: 259].

A review of the baseline medical histories taken in EarlyACTID identified that, in contrast to NICE guidelines, many patients were not given the opportunity to make any lifestyle changes before commencing on oral hypoglycaemic agents within one month of diagnosis [51]. As such, those in the intervention arms of the trial (receiving intensive diet, or diet and exercise support), were afforded the first opportunity to set dietary goals, such as moderating fat intake or increasing the amount of fruit and vegetables that they ate [52]. This led Malpass et al. to conclude that the first year post-diagnosis is a ‘crucial period of time’ where patients can be supported to modify their lifestyles in ways that are necessary to develop a sense of control ‘over time’ [28] (Italics added).

This is reflected in the levels of support described by some of the participants in the usual care study who, without adequate support and information, developed their own interpretation of the relationship between their experience of diabetes symptoms and service provision over time.

When T2DM is first diagnosed, people often make intuitive inferences about the severity of their condition, based upon common sense notions of both symptoms and locations of service delivery:

‘Mary, for example, gave the very strong impression in all of her interviews that there was little, if anything, about the health services with which she had had contact that had indicated to her that she had a potentially serious disease…Particularly striking in her interviews, however, is the assumptions she had made about why all of her care had remained in general practice. Mary, like most other patients who took part in the study, perceived hospitals as places where “you really get looked after” (Ellen) because they are frequented by diabetes consultants (i.e. specialists) who provide “the ultimate knowledge” (Andy). Accordingly, not receiving a hospital referral and/or having to wait for what was perceived as a long time for an appointment to come through were commonly interpreted by patients as indicating that they could not have a potentially serious disease.’ [Author quote, [39]: 1428–9]

Similar interpretations were made when participants were prescribed equipment (e.g. blood glucose meters or urine testing sticks) and, in addition, some of those conducting urine testing perceived a negative test result as indicative of an absence of diabetes, which could impede subsequent self- management [38].

The intervention studies provided a supportive environment in which participants could understand and develop techniques for monitoring and managing their condition which fitted in with their lives, and which they could subsequently try out in the real world setting and discuss with trial staff. In contrast, participants receiving usual care often lacked insight into their condition, which ameliorated both the understanding and confidence required to effectively self-manage, and led to many disengaging with care providers on all but a superficial level.

### Timeliness of support

We developed a third order concept around the notion of timeliness of support: the provision of appropriate support and information, often repeatedly, tailored to the current needs and preferences of the patient, and their subjective understanding of the progression of their condition. Without such timely support, patients are unable neither to be stakeholders in decision making nor to be empowered to make changes. While those in the intervention studies received frequent opportunities for re-engagement with professionals and reinforcement of diabetes knowledge, those in the usual care study were left largely to fend for themselves.

Participants receiving the educational intervention (DALY) identified the course as providing optimal conditions for ‘pressurising’ them to take more notice of their health adding that ‘the protected time for learning is very important’ [Participant quotes, [25]]. They emphasised the significance of ‘real life’ and the need for practitioners to acknowledge that compromise was required for them to conform to treatment regimens [Participant quote, [26]]. The study authors conclude that timely education can lead people to re-evaluate their perception of diabetes as a ‘mild’ disease, and that this change can allow them to view diabetes as ‘integral’ to themselves, and therefore less of a ‘threat’ to manage [Author quote, [26]].

Quantitative findings suggest that the DALY intervention produced sustained improvement in both illness attitudes and self-monitoring at 12 months, with the researchers concluding that continued reinforcement may be required to sustain behaviour change and notions of self-involvement [27].

In the PACCTS study, one of the participants was explicit about the impact of having a supportive tele-carer work with them to identify problems as they arise and negotiate strategies at their own pace:

‘Because I am conscious of the fact that I have to give those figures to somebody and it has been explained to me although I do not dwell on it, the implications if I do not control my levels the fact is I am susceptible to strokes, etc. The underlying factors of diabetes, I do not like to think about it but I have been made aware through calls and general conversation… I really, really love chocolate. I could eat four bars in the morning, and I am not saying I do not touch it but, I am more conscious of the damage.’ [Respondent quote, [32]: 224].

The authors suggest that adopting a ‘diabetic identity’ is fundamental to effective self-management; although they emphasise that this emerging and evolving identity is not one based on ‘adherence’, but rather an ‘enhanced self-agency…albeit potentially constrained by [one’s] own socio-economic circumstances, demographic profile and other ill-health or mobility restrictions’ [Author quote, [26]: 280] - illustrated by this account:

‘I don’t think I would be here if I had carried on the way I was…. Within 12 months, I was down to low numbers and now, I am in the 7 s. I have cut the drinking down by 70%… The information, changing over time, has improved me and I think it is invaluable. The call centre has met all my expectations even gone above them…’ [Participant quote, [33]: 277, Reviewer emphasis]

Both of these quotes emphasise that agency is facilitated by regular, repeated and timely contact [33].

EarlyACTID participants also identified that confronting the reality of their diagnosis was a motivator for change: ‘I’m in control because fear made me control my diabetes’, and ‘I don’t want to end up on insulin . . . if I can maintain this level of health I will be happy . . . I want to avoid even going on tablets’ [Participant quotes, [28]: 260]. However, a small number countered that making multiple lifestyle changes could be difficult, with two men concluding that increasing the amount of exercise, while reducing portion sizes, was counterproductive; while a female participant struggled to make changes without the support of staff or family [28]. This emphasises that change needs to be at a pace that is suitable for an individual, and taking into social roles, in order to achieve sustained behaviour change.

In the absence of on-going support, providing one’s own interpretation can become an enduring aspect of living with diabetes, with implications for both self-monitoring and effective management. Those in the usual care study articulated a tension between wanting to receive knowledge in the early days after diagnosis while, at the same time, being unable or unwilling to articulate their concerns. After diagnosis, some respondents were mindful of taking up the time of health professionals:

‘It takes that long to get an appointment with the GP you feel silly going in and saying er ‘Should I reduce my Metformin?’ and he’ll say ‘Nope’.’

‘You go to your GP and you’re aware all the time that you’ve got five minutes to get this over and get out, and that’s at the back of your mind. You know, and I’m sitting there thinking ‘I’ve no got to bother her today with all these questions’…I’m thinking ‘oh well some poor soul behind me could have cancer or whatever’, you know.’ [Respondent quotes, [40]: 1248–9]

Others identified that appointments did not always coincide with their information needs:

‘As I say as time goes on you get more and more used to it and you get more and more able to deal with it yourself. But initially it really is, erm certainly was for me, a real – I was in shock. And inevitably that asks…begs many questions that you want to ask and you’ve got to kind of put in a request to see y’know, well how, wait a minute, a request to see somebody, no, hang on, why can’t I just- why can’t I just have an answer to my question, simple little thing, that’ll put my mind at rest. [Respondent quote, [40]: 1249]

Over time, some participants were more open about the fear associated with ‘knowing too much’ or adopting what they perceive as a ‘diabetic identity’:

Eric: Erm I think I know enough erm but erm I don’t feel that y’know at the moment I-I don’t need, er,don’t want others to talk to me about diabetes. I think that might suggest that I’m becoming obsessional about the damned thing and I-I don’t know if I want … if you’re sort of searching out people or organisations that are talking about diabetes all the time, you sort of become a diabetic person and erm well you’re somebody else then.

Jennifer: No, I read quite a bit about it y’know on the leaflets and that. And sometimes I often think there’s a book that they advertise in all the newspapers and I think “I’m going to send away for that” but sometimes I think you can know too much. So I’ve never done it.

(I: What do you mean like in terms of knowing too much? Like because it might worry you more?)

Jennifer: Yeah, yes. That’s exactly what I mean.[Respondent quotes. [40]: 1431]

Furthermore, most patients considered that their need for prompt information and reassurance would attenuate over time [39], which suggests that, in the absence of appropriate information, some people are unable to appreciate the chronic nature of their diabetes and anticipate their future needs.

The benefit of exploring longitudinal studies is apparent in subsequent interviews, where participants describe the cessation of self-monitoring, although ‘none of the participants reported having been explicitly told by health professionals to stop self-monitoring, nor had they received additional education about self-monitoring after the first year following diagnosis’ [Author quote, [42]: 494]. A particular note is made of ‘older and less well educated participants’, who the authors believe are particularly vulnerable to negative attitudes of health professionals, and who may continue the process of monitoring without fully engaging in it, for the benefit of the health professional, rather than the patient:

‘Four checks a week, I do. But I write it down, and that’s as far as it goes’ [Participant quote, [42]: 495]

With time, this lack of engagement may extend from self-monitoring to self-management. With regard to the role of exercise, ‘few participants acknowledged that physical exercise overall was fundamental to their diabetes self-care’ [Author quote, 11: 572], and several emphasised the perceived lack of interest expressed by their health professionals:

‘Well they’ll ask, y’know, what exercise you get … but they haven’t said “Oh I think you should be walking twice as far” no, nothing like that.’ [Participant quote, 11:572]

Likewise, in relation to medicine taking, ‘few respondents claimed to be fully adherent, highlighting forgetfulness as the central reason for this’ [Author quote, [44]: 493]. Forgetfulness was common for asymptomatic patients where diabetes was not ‘at the forefront of your mind’, for those with busy lives who forgot to take their medication with them, and those with multiple co-morbidities who stressed that, ‘if you take a lot of tablets, you’ve no idea when you’ve taken them, and what you’ve taken’ [Participant quotes, [44]: 493].

The intervention studies provided structured opportunities for participants to ask questions and to be provided with timely and appropriate information. In contrast, participants in the usual care study describe a cascade of missed opportunities.

### Empowerment

The final third order concept was developed around empowerment: the willingness and ability of people with diabetes to self-manage in order to achieve purposive and meaningful behavior change strategies. Participants in the intervention studies were often able to claim empowerment as a consequence of the support and information that they received, although not all patients were willing or able to self-manage. The study of usual care illustrated that when patients are in receipt of standard healthcare, diabetes related ‘quality of life’ may also be mediated by one’s orientation or perspective.

Participants in the DALY study valued collaborative learning:

‘I’ve learnt about other people’s ideas, other people’s problems and you find that you are not on your own. You can learn how they are overcoming the problems.’ [Participant quotes, [26]: 202].

Accepting a ‘diabetic identity’ and sharing experiences ‘between equals’ facilitated a ‘group empathy’ which enabled participants to ‘analyse motives for their current behaviour’ and provide ‘opportunities for them to learn new skills in relation to self-managing their diabetes.’ [Author quotes, [26]: 202]:

‘I am able to bend more now. I no longer find it [diabetes] a nuisance.’ [Participant quotes, [26]: 202].

A similar confidence developed among participants in the EarlyACTID trial, who were able to titrate diet and exercise in such a way that that self-management enabled both blood glucose control and quality of life:

‘For example, Wayne (DPAI) tried hard to follow dietary recommendations but enjoyed drinking alcohol and eating out. Both were key to his friendship and relationship building. To counterbalance the effects of these two behaviours, Wayne would “work a bit harder in the gym the next day”.’ [Author quote, [26]: 260]

While participants in both of the above educational intervention studies acknowledged that motivation was needed to supplement their learning, those who were supported to improve their level of physical activity were able to identify that a cycle of behaviour change had been set in place, such that improvements to their physical and mental health encouraged them to eat healthy food and persist in their self-management:

‘I always feel better when I come back (from the gym), I always feel I’ve got more energy . . . when you’re exercising you’re saying ‘I’m doing all this, I ought to cut back a bit’ (laughs).’

‘Having gone, exercised and come back, you feel really rejuvenated, and I think it spurs you on to keep motivated.’ [Participant quotes, [28]: 260]

Thus physical activity could be viewed as both a motivator for, and integral to, diabetes self-management.

At twelve months, PACCT participants with a baseline HbA1c greater than 7% achieved significant improvement in glycaemic control [34] and high levels of satisfaction [35]; while follow-up at three years identified a continuing significant reduction of HbA1c attributed to the intervention, without additional pharmacological means in a sample drawn from a socio-economically deprived urban community [36]. Despite the PACCTS intervention being well received by both participants and health care providers, a subsequent economic evaluation of PACCTs found it to be borderline cost effective [37].

However, even within a supportive trial environment, some participants remained unempowered when it came to self-management. In the tele-support study (PACCTS), the authors describe one man who, despite having his knowledge enhanced, was unable to translate that awareness into effective control after two years of study participation:

‘He seems to have found the calls somewhat irritating: they always ask me the same question, ‘are you eating say this, this? ‘ It’s always the same. But, he remarked, if I’d had diabetes for a year I could have understood it but this is fifteen years, well, three years of this now and I know what they are going to say. He feels that he has his diabetes under control… He has not really changed the way he eats, except in relation to the amount of sugar…’ [Author quotes, [33]: 257]

Furthermore, Cooper et al. conclude that the ‘low uptake of patient education may not just reflect a cultural climate that promotes dependency’, which they attribute to a bureaucratic health care system in the UK and health professionals who lack specialist knowledge, ‘it may also reflect patients’ desires to continue with their passive role’ [[25]: 204–5].

In contrast to notions of a ‘passive’ patient, the study of usual care unpacked the dynamic relationships between patient perspectives and behaviour, in the absence behaviour change interventions. By exploring changes in causal accounts over four years, Lawton et al. contend that treatment experiences mediate respondents’ disease perceptions [43]. Whereas ‘Ellen’ maintained her causal account of her diabetes being due to her poor diet over time, ‘Fiona’ amended her account from having a dietary cause to being hereditary, on the assumption that ‘even my son’s got it now… it must be hereditary’ [Participant quote, [43]: 51]. In contrast ‘Graham’ begins with a hereditary account of his diabetes, and later acknowledges that he ‘made a pig of myself and put on a lot of weight’ [Participant quote, [43]: 51]. Thus, Lawton et al. suggest that ‘causation accounts may be informed by, and revised in light of, the perceived efficacy of treatments’ [Author quote, [43]: 51].

Importantly, these ‘causal accounts’ do not ‘simply convey respondents’ “beliefs” about disease causation… they also serve a communicative or interactional role… [and can] be used as vehicles to rationalise, legitimate and/or enable particular approaches to T2DM self-management.’ [Author quote, [43]: 52]. While ‘Mary’ attributed her diabetes to ‘bad living’, by also emphasising that her mother had lived a healthy life but died of a stroke, she provides legitimacy for not engaging in self-management activities [[43]: 53]. A legitimising account is also present in this respondent’s account, where medicine taking is viewed as controlling blood glucose to the detriment of quality of life:

‘By his third interview, Callum had “compared notes” with work colleague with T2DM who had recently moved on to insulin, and “seemed to control things a lot better”. By virtue of being able to titrate her insulin doses, this colleague appeared to have the freedom to eat and drink what she wanted, a freedom which Callum professed to desire. At this point, Callum stopped talking about being able to control his own diabetes with tablets and diet, suggesting that “sooner or later, it’s going to become an insulin issue”. He also ceased to blame any “spikes” recorded through SMBG on his continued snacking. Instead, he attributed them to “the tablets no longer working”, and used this to justify bringing forward his appointment with his consultant and negotiating a move to insulin: “I eventually convinced them I was ready for it”’ [Author quote, [43]: 53].

By re-framing his orientation, this participant is able to legitimise his transition to commencing insulin as a strategic form of self-management that will improve his quality of life.

Participants in the usual care study were able to develop causal accounts that could inhibit their sense of agency and legitimise their inability to self-manage. Furthermore, the development of lay causal narratives and the negation of a ‘diabetic identity’ (resulting from lack of timely information and support) enabled some respondents to absolve themselves of any responsibility for their diabetes and inhibit subsequent behaviour change. In contrast, with the support and information integral to intervention studies, trial participants were better able to achieve empowerment. This enabled participants to develop flexible diabetes management strategies that facilitate (rather than inhibit) quality of life and long term medical outcomes (including blood glucose and weight control.

### Summary

Having identified three interlocking third order constructs (Patient as stakeholder, Timeliness of support, and Empowerment, that can be positively or negatively reinforcing), the synthesis substantiated a line of argument that stated that for self-management strategies to be effective, people with diabetes require a sense of ownership of the management of their disease. This can be fostered through the timely provision of information and advice that acknowledges and accounts for their individual circumstances (e.g. disease duration, and prior experience of diabetes management).

‘I feel better certainly. I am not getting infections […] Before, I used to get thrush and infection after infection because my blood sugars were out of control. I take more care in things like having my feet done. I do not know, I just feel healthy […] I am sure that I will continue to follow the advice.’ [Participant quote at 3 years; 32: 224]

In contrast, strategies can be undermined when health professionals do not take account of patients’ beliefs and values, or when self-management is limited to monitoring rather than the means to moderate ‘treatments’, (e.g. when advice is generic rather than tailored to an individual’s support needs). While it is acknowledged that not all people with diabetes are willing or able to self-manage their condition, a flexible regimen is associated with a balanced approach to self-management that facilitates quality of life; while a self-management strategy that is perceived as encroaching upon quality of life (e.g. by inhibiting participation in social activity, such as family meals) has little or no positive impact:

‘I walk out and into the pigeon loft at the back door, over to the shop for my cigarette papers’ [Participant quote at 4 years; 11: 573]

## Discussion

Noblit and Hare contend that the objective of an interpretive synthesis is either to make the obvious obvious, make the obvious dubious, or make the hidden obvious [18]. While it has long been recognised that patients with chronic illness require enduring support to effectively self-manage their condition [12], this longitudinal qualitative synthesis has demonstrated that this is still not routine practice, and that this omission may have a cumulative deficit for people with T2DM. These findings suggest that, in the absence of timely support and advice, the construction of elaborate lay models over time may have a self-protective effect, which can mitigate a sense of failure and liability. It is incredibly difficult for someone with an enduring and unchallenged hidden causal account that minimises behavioral causes of T2DM and/or the validity of self-management, to become a confident and flexible self-manager, as this requires an acknowledgement of one’s accountability.

Building upon existing retrospective cross-sectional accounts, the usual care study claims that if people do not acquire a stake in their diabetes management shortly after diagnosis, or they lose that stake due to lack of support, this can have an immediate impact upon how they frame ‘their diabetes’ which may subsequently negate their ability to make informed decisions and choices [9, 10]. While the on-going information and support, which are central to the underpinning philosophies of the intervention studies, foster understanding and confidence and ultimately self-efficacy, the findings of the study of usual care identify that unsupported self-management can lead to people with diabetes not fully engaging with resources and behaviours, the result of which may be detrimental to their quality of life. Additionally, while the usual care study identified that the empowerment required for the effective management of quality of life and biomarkers can be mediated by a person’s orientation or perspective, even resource intensive interventions cannot guarantee that participants will willingly embrace self-management. By extending the time-frame of papers included in this synthesis (e.g. those with repeat data collection over twelve months, rather than one off interview studies), we are able to build upon the findings of existing syntheses. The ability to achieve balance [12, 13] may be mediated by cycles of behavior change, such that with adequate support a positive feedback mechanism can develop in relation to diet and exercise which may facilitate both quality of life and control of biomarkers, while unsupported self-management can inhibit the effective management of Hba1c, as the perceived burden on one’s quality of life may be too great.

These findings suggest that people with diabetes both value and profit from ongoing support and information, from both health professionals and peers [53, 54], when it is reciprocal and tailored to their own needs. The challenge is to deliver continuity of individualised care in the context of current changes to the healthcare system in the UK and elsewhere. The emphasis should be on small patient-centered goals, such as weight-loss or portion control, that are achievable, rather than the prioritisation of biomarkers, which some people may perceive as unachievable and burdensome. Not all people with diabetes have the propensity or ability to self-manage, and following Noblit and Hare’s assertion that the purpose of comparative translation is ‘not to achieve closure, but to enable discourse’ [18], greater understanding is now required of how health professionals may both inhibit and facilitate self-management in specific populations (such as the elderly or those with multiple co-morbidities) who may have additional needs that interact with any diabetes specific information and support. There is now a need to facilitate on-going open dialogue in usual practice, in order to achieve sustainable change in diabetes self-management.

This paper has demonstrated that it is possible to synthesise longitudinal qualitative papers in order to identify strategies for the long term management of T2DM. Although data from only four patient samples were synthesised, this rich a dataset represents over one hundred interviews and ten focus groups, collected over a four year period. This is in keeping with the amount of data synthesised in previous meta-ethnographies [13].

Combining qualitative papers associated with different objectives and methodological approaches is not without challenge [48, 50]. The impact of the ideological and theoretical perspectives inherent in the study of usual care, and their impact on data collection and analysis were not always clear, and we were unable to assess the sustainability of change that could be attributed to educational interventions beyond three years. The authors of the intervention studies identified that the supportive trial environment may have enhanced the positive impact of the interventions [53], We contend that the comparison between the two types of studies illuminated the information and support that were missing from usual care, and which are necessary for people with diabetes to successfully self-manage. The supportive trial environments allowed patients to test out and discuss new monitoring and management strategies. Both sets of papers suggest that this does not routinely happen in ‘usual care’. Patients describe an inability to articulate or willingness to discuss their concerns early on in the disease trajectory, with few, if any, subsequent opportunities to subsequently ask key questions. This negation may lead to some people never fully grasping the nature, severity, or progressive nature of their condition, or which monitoring and management practices are most appropriate.

All four studies acknowledge that the qualitative study samples may have been self-selected [54]. Furthermore, most of the participants in the studies were white British; although we anticipate that research currently being conducted by Greenhalgh et al. [55] could further illuminate the mediating roles of migration and culture [56]. However, we were able to develop a line of argument that for self-management strategies to be sustainable beyond the duration of clinical trials, patients require timely information and support, a sense of having a role in their management that is appropriate for their beliefs and perceptions, and an overall sense of empowerment in managing their diabetes in relation to other aspects of their life.

## Conclusions

This synthesis has explored how patients give meaning to the experiences of interventions for T2DM and subsequent attempts to balance biomarkers with quality of life in the long term. People with T2DM both construct and draw upon causal accounts as a resource, and a means to counter their inability to balance medical outcomes and quality of life. These accounts can be mediated by the provision of timely and tailored information and support over time, which can allow people to develop a flexible regimen that can facilitate both quality of life and medical outcomes.

## Abbreviations

T2DM: Type 2 diabetes mellitus.
